# A Systematic Quantitative Determination of the Antimicrobial Efficacy of Grape Seed Extract against Foodborne Bacterial Pathogens

**DOI:** 10.3390/foods12050929

**Published:** 2023-02-22

**Authors:** Melina Kitsiou, Lisa Purk, Jorge Gutierrez-Merino, Kimon Andreas Karatzas, Oleksiy V. Klymenko, Eirini Velliou

**Affiliations:** 1 School of Chemistry and Chemical Engineering, University of Surrey, Guildford GU2 7XH, UK; 2 Centre for 3D Models of Health and Disease, Division of Surgery and Interventional Science, University College London, London W1W 7TY, UK; 3 School of Biosciences and Medicine, University of Surrey, Guildford GU2 7XH, UK; 4 Department of Food and Nutritional Sciences, University of Reading, Reading RG6 6AD, UK

**Keywords:** natural antimicrobials, grape seed extract, microbial inactivation, stress response, *Listeria*, *E. coli*, *Salmonella*

## Abstract

Concerns regarding the role of antimicrobial resistance (AMR) in disease outbreaks are growing due to the excessive use of antibiotics. Moreover, consumers are demanding food products that are minimally processed and produced in a sustainable way, without the use of chemical preservatives or antibiotics. Grape seed extract (GSE) is isolated from wine industry waste and is an interesting source of natural antimicrobials, especially when aiming to increase sustainable processing. The aim of this study was to obtain a systematic understanding of the microbial inactivation efficacy/potential of GSE against *Listeria monocytogenes* (Gram-positive), *Escherichia coli* and *Salmonella* Typhimurium (Gram-negative) in an in vitro model system. More specifically, for *L. monocytogenes*, the effects of the initial inoculum concentration, bacterial growth phase and absence of the environmental stress response regulon (SigB) on the GSE microbial inactivation potential were investigated. In general, GSE was found to be highly effective at inactivating *L. monocytogenes*, with higher inactivation achieved for higher GSE concentrations and lower initial inoculum levels. Generally, stationary phase cells were more resistant/tolerant to GSE as compared to exponential phase cells (for the same inoculum level). Additionally, SigB appears to play an important role in the resistance of *L. monocytogenes* to GSE. The Gram-negative bacteria under study (*E. coli* and *S*. Typhimurium) were less susceptible to GSE as compared to *L. monocytogenes*. Our findings provide a quantitative and mechanistic understanding of the impact of GSE on the microbial dynamics of foodborne pathogens, assisting in the more systematic design of natural antimicrobial-based strategies for sustainable food safety.

## 1. Introduction

Food consumption trends have changed drastically over the last years. Consumers are now demanding products that are minimally processed and as free as possible from chemical preservatives and antibiotics [1,2,3]. Additionally, concerns regarding the role of antimicrobial resistance (AMR) in disease outbreaks are growing [4]. Pathogenic bacteria that are resistant to antimicrobials can infect people via multiple routes due to their resistance and higher survival likelihood in multiple environments. More specifically, contamination routes can be through food, water, and environmental contaminants as well as through direct animal contact. In addition, infections caused by resistant bacteria could be difficult to treat, necessitating administration of multiple antimicrobials [5]. The main factor driving AMR is the excessive use of antimicrobials, i.e., tetracyclines, penicillin, and sulphonamides are the most commonly used drugs in animals, in livestock, crops and along the production chain [6,7]. Nevertheless, reducing the use of (commonly used) antimicrobials through finding novel control strategies is a key factor for managing the upcoming AMR crisis [8,9]. Moreover, as a result of climate change, environmental consciousness is more prevalent than in the past, prompting the food industry to adopt a more sustainable approach to the production design [10,11]. Sustainable production could be achieved through the implementation of environmentally friendly processing technologies, the replacement of traditional energy-consuming heat-processing technologies, a reduction in waste and the valorisation of food by-products within the food industry [10,11].

The possibility of using natural antimicrobials to replace chemical preservatives and the extensive use of antibiotics has been one of the major goals of the food industry [12]. By using natural antimicrobial components, health risks associated with chemical additives and AMR could be reduced [13,14,15].

Fruit and vegetable by-products are particularly interesting as sources of natural antimicrobials, especially when aiming at increasing sustainable processing in the food industry. Valorising waste of a food processing line which otherwise would have been disposed provides additional motivation for researching their antimicrobial activity as it contributes to the reduction in food waste [16]. Fruit and vegetable by-products such as peels originating from lemon, orange, banana, pomegranate, potato, grape or apple and seeds from mango, grape and avocado have already showcased promising antimicrobial activity. This antimicrobial activity has been attributed to the presence of different groups of polyphenols depending on the fruit by-product [17,18,19,20,21,22,23,24].

Grapes are one of the world’s most valuable crops. The global annual production reaches 25 million tonnes. Grape by-products are generated from grape processing to produce wine and juice. They represent approximately 20% of the total weight of the grape, making their disposal a challenging problem for wineries and the grape juice industry [25,26]. Grape by-products consist of the skins, seeds and, in some cases, the stems of the fruit [27,28]. Some studies have indicated that grape seed extract (GSE) contains the highest concentration of antioxidant and antimicrobial compounds, such as polyphenols, in comparison with other grape by-products [26,29,30,31]. GSE has also been generally recognised as a safe (GRAS) food additive allowing its wider use as an antioxidant. However, to date, GSE is not commonly utilised as an antimicrobial agent [32,33]. The concentration of polyphenols in grape pomace is affected by many parameters, namely, grape variety, climate at the place of origin and time of harvesting [34,35]. For example, grape pomace from the Syrah variety has been reported to have lower phenolic content than the Merlot variety with 1013 and 2022 mg gallic acid equivalent (GAE)/100 g, respectively. [36]. In addition, the method of extraction such as the type of solvent in solvent-based extractions or operational parameters of other methods such as ultrasound, pulsed electric fields, hyper-pressure, used for assisting the extraction, can have a major influence on the composition and concentration of the final extraction product [37,38,39].

Several studies have investigated in depth the antioxidant activity and possible methods to extract the bioactive compounds from grape by-products [27,37,40,41]. Additionally, it has been shown that grape pomace extracts have antimicrobial properties [27,34,42,43,44,45,46]. The bacterial inactivation by GSE has been attributed to various mechanisms of action such as the ability of polyphenols to penetrate bacterial cell walls and the antibacterial activity of tannins to inactivate extracellular enzymes. In addition, GSE is able to form complexes with metal ions resulting in their depletion from the bacterial environment. [47,48]. Metal ions are necessary for pathogenic bacteria to preserve protein structure and function and maintain their pathogenicity; therefore, their blockage by GSEs leads to bacterial damage [49].

Most studies have focused on the extraction of polyphenols from locally produced grape pomaces and on whether these extracts have a microbial inhibitory effect. More specifically, those studies have been conducted in different parts of the world, i.e., the USA, Turkey, Croatia, Portugal, Brazil, and Spain and on various microorganisms, i.e., *Listeria* spp., *Bacillus* spp., *E. coli*, *Staphylococcus aureus*, *Salmonella enterica*, and *Pseudomonas aeruginosa* [27,34,42,43,44,45]. However, in those studies, there was no systematic quantitative monitoring of the microbial dynamics/kinetics over time. To determine the microbial inhibitory effect, the most common methods used by all of the above studies are the agar/disk diffusion test and the minimum inhibitory concertation [50]. These tests are very useful to rapidly confirm the inhibitory activity of a substance but provide no information regarding the exact quantitative inactivation dynamics of the microorganism under research. Consequently, it is not possible to use only such results for the robust design of an industrial or clinical treatment process.

To the best of our knowledge, there are very limited studies quantifying the antimicrobial activity of GSEs, and those studies were mostly conducted on specific food products, mainly in meat products, making the extrapolation of their findings to other products challenging. GSEs have showcased good antimicrobial activity against Gram-positive bacteria. For example, the agar diffusion test has shown that GSEs were effective against *L. monocytogenes*, *Bacillus cereus*, *Enterococcus faecalis*, *Enterococcus faecium*, *S. aureus*, *Staphylococcus epidermidis* and *Mycobacterium smegmatis* [42,47,51] Additionally, the anti-listerial activity of the GSE has been shown by some studies carried out in nutrient broth, meat products (turkey Frankfurters and ground and cooked beef) and/or seafood (shrimps) [29,52,53,54,55] However, results are contradictive regarding the inactivation of Gram-negative bacteria [40,47,55]. For instance, Baydar et al., (2006) observed that 1% GSE inhibited *E. coli* and *S.* Typhimurium in an agar diffusion test. However, in a study by Corrales et al., (2009) using the same methodology, no inhibition was observed for those bacteria [47].

In studies where nutrient broths were used instead of an actual food product, the microbial concentration was measured mostly once post-treatment [48,56,57]. *Systematic monitoring of the post-treatment bacterial dynamics can help observe potential inactivation trends and/or potential post-treatment bacterial re-growth and/or resistance, especially in the early stages of bacterial growth.* Lastly, the majority of the published papers used bacterial cultures in the stationary phase of growth in which the cells are more resistant to antimicrobials [29,32,52,58]. *This might not completely represent the reality, i.e., the growth phase of microorganism after food contamination occurs.* For example, if only a few hours have elapsed after the food contamination event, the microbial cells could be in the exponential growth phase. During the exponential growth phase, the stress response of the cells is reported to be different, as certain transcriptional regulators that control gene expression do not reach their peak until the stationary growth phase [59,60]. One of these regulons is the alternative sigma factor, encoded by *sigB* in Gram-positive bacteria such as *L. monocytogenes*. SigB is altered in response to varying environmental conditions and it plays a major role in the stress adaptation of *L. monocytogenes* in multiple types of stress such as thermal, acid or osmotic stress [60,61,62]. In addition, the antimicrobial resistance to certain bacteriocins such as nisin and lacticin 3147 is affected by SigB [61,63]. However, there are no studies reporting the role of SigB in the presence of natural antimicrobials and GSE in particular.

Overall, despite their great potential, in order to use grape by-products effectively as natural antimicrobials on a larger scale, additional research is required. The purpose of this study is to examine the systematic microbial dynamics of Gram-negative (*E. coli* and *S.* Typhimurium) and Gram-positive (*L. monocytogenes*) foodborne pathogens and their stress mutants in different growth phases and at different concentrations (inoculum levels), as a function of GSE concentration, using a liquid in vitro model system, i.e., Tryptone Soy Broth with Yeast Extract (TSBYE), which is a well-defined, reproducible, nutrient-rich medium used extensively in the literature for fundamental microbial studies [50,52,64,65,66,67,68,69]. Such a systematic comparative study will shed light on the impact of the type of bacteria, their growth phase, inoculum concentration and (environmental) stress state on the bacteriostatic and bactericidal effect of GSE. This knowledge can lead to the more accurate incorporation of GSE in food processing for microbial inactivation purposes, individually or in combination with other novel technologies, enabling the attainment of sustainable food processing.

## 2. Materials and Methods

### 2.1. Inoculum Preparation

The following microbial pathogens were studied: (i) *Listeria monocytogenes*, (ii) *Escherichia coli*, (iii) *Salmonella enterica* serovar Typhimurium. These bacteria were selected as they account for the majority of foodborne illnesses. Furthermore, *L. monocytogenes* in particular has the highest reported fatality rate [70]. Additionally, a mutant strain of *L. monocytogenes* was used to determine the significance of its environmental stress response and/or survival in presence of grape seed extract (GSE). Stock cultures of *L. monocytogenes* 10403S *WT, L. monocytogenes* 10403S Δ*sigB*, *E. coli* MG1655 *and S.* Typhimurium ATCC 14028 were stored in Tryptone Soy Broth (TSB, Oxoid Ltd., UK) supplemented with 15% glycerol at −80 °C. The inoculum preparation took place as previously described [3,71,72,73,74,75,76,77]. More specifically, a loopful of thawed culture was inoculated in 20 mL TSB supplemented with 0.6% of Yeast Extract (TSBYE) and cultured in a shaking incubator at 37 °C and 175 rpm for 9.5 h. Thereafter, 20 μL was transferred in 20 mL TSBYE and cultured for another 15 h until the early stationary phase was reached (10^9^ CFU/mL). For the experiments where cells in the mid-exponential phase were used, cells were further cultured (for another growth cycle) at 37 °C and 175 rpm until the mid-exponential phase was reached (10^5^ CFU/mL).

### 2.2. Preparation of GSE Solution

The present study used commercially available GSEs (Batch No. 000128872, Bulk, Colchester, UK). According to the manufacturer, the powder contains a minimum of 95% oligomeric proanthocyanidins. The GSE powder was dissolved in TSBYE at 1% and 4% *w*/*v* and autoclaved (GSE concentration will be expressed simply as per cent in this manuscript from now onwards). These GSE concentrations were determined based on preliminary tests (results not shown). The autoclaved TSBYE-GSE solution was stirred overnight to ensure homogeneity.

### 2.3. Microbial Dynamics in the Presence of GSE

To examine the effect of GSE against the tested pathogenic bacteria, cells (obtained as described in Section 2.1) were appropriately diluted in TSBYE medium and transferred via pipetting to 1% and 4% TSBYE-GSE solutions. Additionally, all bacteria were inoculated in TSBYE without GSE (controls). For *L. monocytogenes*, different initial inoculum concentrations (10^5^ and 10^8^ CFU/mL) and growth phases (stationary and mid-exponential) were studied. Testing different initial cell concentrations reveals if the same GSE concentration would be effective for heavier contaminations. The initial population of *E. coli* and *S.* Typhimurium was approximately 10^5^ CFU/mL and only stationary phase cells were studied, as the inhibitory effect of GSE was much weaker than that for *L. monocytogenes* even for (stress-adapted) stationary phase cells where maximum resistance is expected (see Section 3). All tested parameters can be seen in Table 1.

After inoculation, the samples were kept at 37 °C. The bacterial survival was systematically monitored for up to 24 h post-treatment (at 0, 2, 4, 8, 12, 18, and 24 h) using the plate count method in non-selective agar, i.e., Tryptone Soy Agar supplemented with 0.6% of Yeast Extract (TSAYE, Oxoid Ltd., Basingstoke, UK).

### 2.4. Statistical Analysis

At least two independent experiments with 3 replicate samples were conducted. For each replicate sample, the mean, standard deviation, and standard error were calculated. To compare two mean values, the Student’s t-test was used. For *p* < 0.05, the difference between two unrelated groups was considered statistically significant. In the plots below, the mean value is presented with error bars representing the standard deviation. In cases where the viable cell count was below the detection limit (<10 CFU/mL) the number was set to 1 log CFU/mL. Statistical analysis was performed using Microsoft Excel.

## 3. Results and Discussion

### 3.1. The Impact of GSE on the Microbial Dynamics of L. monocytogenes

In order to perform a systematic study of the impact of grape seed extract (GSE) on the microbial dynamics of *L. monocytogenes* in an in vitro liquid system, different parameters were varied, namely: (i) the concentration of GSE, (ii) the initial inoculum concentration, and (iii) the microbial growth phase. Additionally, the isogenic Δ*sigB* mutant of *L. monocytogenes* was used to explore the potential mechanism of inactivation of GSE on *L. monocytogenes*. As previously mentioned, SigB is a stress gene regulator that is crucial for *L. monocytogenes to* adapt in multiple environmental stresses such as thermal, acid or osmotic stress [60]. Therefore, the absence of SigB might increase the sensitivity of *L. monocytogenes* when treated with GSE.

*To the authors’ best knowledge this is the first study performing a systematic quantitative comparison of the effect of those parameters on the action of GSE against L. monocytogenes.*

#### 3.1.1. The Impact of the Bacterial Initial Inoculum Concentration on the Activity of GSE against *L. monocytogenes*

To investigate the impact of the initial inoculum level on the action of GSE against *L. monocytogenes* 10403S WT, two different initial bacterial concentrations were used, i.e., 10^5^ CFU/mL and 10^8^ CFU/mL, representing different levels of contamination of the in vitro system. Bacteria were (pre-)grown to stationary phase and appropriate dilutions were performed to reach 10^5^ CFU/mL and 10^8^ CFU/mL. Thereafter, the bacterial cells were added in TSBYE containing different concentrations of GSE, i.e., 1% and/or 4%. The GSE concentrations were selected based on previous studies that showed that 1% GSE had inhibitory activity (agar diffusion test) [47,51,55] and on preliminary tests (data not shown).

**Figure 1:**
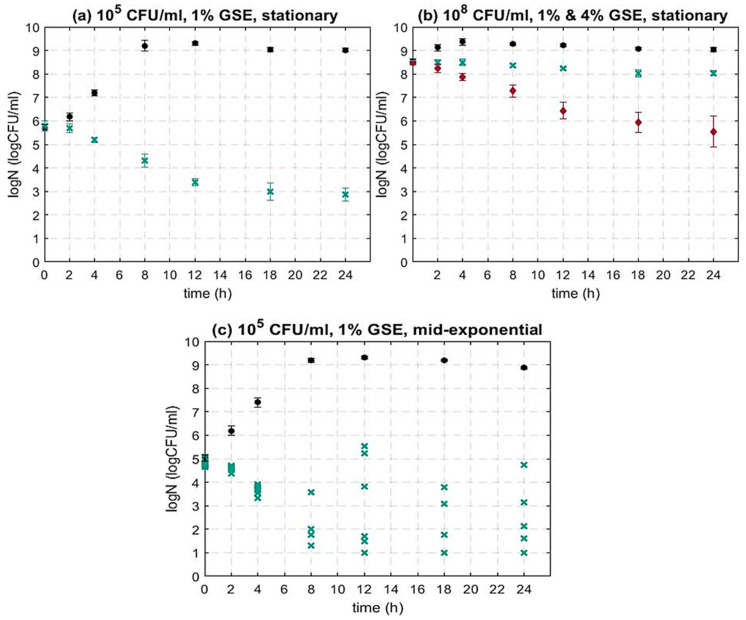
Inactivation dynamics of *L. monocytogenes* 10403S WT at initial inoculum concentration (**a**) 10^5^ CFU/mL, stationary phase cells (**b**) 10^8^ CFU/mL, stationary phase cells (**c**) 10^5^ CFU/mL, mid-exponential phase cells. In all plots, (●) control (w/o GSE), (**×**) 1% GSE. In the (**b**) plot, (♦) 4% GSE. In (**a**,**b**), each time point represents the average of two independent experiments with three technical replicates per experiment. Error bars show standard deviation. In (**c**), all replicates (independent experiments) are presented due to high variation between them.

As can be seen in Figure 1a, 1% GSE caused a significant microbial inactivation for 10^5^ CFU/mL initial inoculum concentration. More specifically, after 8, 12, 18 and 24 h, the inhibition of the treated sample was equal to 1.46, 2.37, 2.76 and 2.94 log CFU/mL, respectively. In addition, after 24 h, the difference between the treated sample and the control sample was equal to 6.24 log CFU/mL. As the inactivation of *L. monocytogenes* (10^5^ CFU/mL) by 1% GSE was substantial (~3 log CFU/mL after 24 h), the higher concentration of 4% GSE was not applied for the initial concentration of 10^5^ CFU/mL.

At a higher initial inoculum concentration (10^8^ CFU/mL), 1% GSE was less effective against *L. monocytogenes* (Figure 1b). More specifically, the observed trend shows a bacteriostatic rather than a bactericidal effect. However, when the GSE concentration was increased to 4%, a substantial decrease in bacterial cell concentration was seen. More specifically, the inhibitory activity followed a similar trend to the inactivation of *L. monocytogenes* at 10^5^ CFU/mL challenged by 1% GSE (Figure 1a). After 8, 12, 18 and 24 h, the inhibition of the treated sample was equal to 1.27, 2.08, 2.60 and 2.93 log CFU/mL, respectively. These findings indicate that the inoculum concentration has a significant impact on the inactivation efficacy of GSE. Generally, as previously stated, studies investigating the impact of GSE against *L. monocytogenes* are limited and most are food-specific. To the best of our knowledge, there is no study altering the initial microbial concentration, and only some studies alter the GSE concentration. For example, Ahn et al. (2004) showed, for an initial microbial load of 10^4^ CFU/mL in liquid nutrient media (TSBYE), that the cell concentration of *L. monocytogenes* was below the detection limit after 16 h of treatment with 0.6% GSE. When the GSE concentration increased to 1%, *L. monocytogenes* was completely inhibited after 8 h of GSE treatment [54]. Additionally, Sivarooban et al. (2007) observed a reduction in *L. monocytogenes* of 2 log CFU/mL after 24 h. The cells were challenged with 1% GSE in liquid nutrient medium (TSBYE) and the initial load was slightly higher (approx. 5 × 10^6^ CFU/mL) [52].

Overall, these findings show that the amount of GSE and the microbial concentration in the sample affect the inactivation potential of GSE. Hence, the amount of GSE that could be used for industrial or clinical microbial inactivation purposes needs to be proportional to the contamination in the sample.

#### 3.1.2. The Impact of the Bacterial Growth Phase on the Activity of GSE against *L. monocytogenes*

Most studies investigating the inhibitory activity of natural antimicrobials use cells in the stationary growth phase [29,32,52]. However, contaminations in the food industry can occur at any stage of food processing; therefore, bacterial cells could exist in food products at different growth phases. As previously mentioned, stationary phase cells usually have a fully developed response to environmental stresses making them more tolerant to the exposure to various treatments including natural antimicrobials [58,59,78]. For this reason, it is highly important to study the antimicrobial activity of GSE in different growth phases of bacteria. To examine the effect of GSE in different growth phases (further to stationary phase cells studied in Section 3.1.1), mid-exponential phase cells (10^5^ CFU/mL) were inoculated in TSBYE containing 1% GSE. This GSE concentration was selected based on our previous findings (Section 3.1.1), as we have seen that 1% GSE concentration caused substantial cell inhibition, when the initial inoculum concentration of stationary grown cells was 10^5^ CFU/mL (Figure 1a).

As can be seen in Figure 1, generally, 1% GSE resulted in the microbial inactivation of *L. monocytogenes* for both stationary and mid-exponential phase cells. Furthermore, as can be seen in Figure 1c, the microbial population post-GSE treatment was mostly lower in mid-exponential phase cells as compared to stationary phase cells (Figure 1a), i.e., a greater inactivation was achieved. The literature suggests that cells in the mid-exponential growth phase can be more sensitive to different treatments in comparison with cells in the stationary phase [58,78]. This can be explained by the fact that once a cell enters the stationary phase, the cell’s morphology, physiological state and gene expression alter substantially. The cells become smaller and rounder and the thickness of the peptidoglycan layer of the cell membrane increases. The alternative sigma factors are more active during the stationary phase, and for this reason, the characteristic proteins required for the bacterial survival are generated mostly during this phase [79]. Hence, it is expected that cells are more sensitive to harsh environmental conditions in the exponential phase as compared to the stationary phase of growth. To the best of our knowledge, there are no other studies comparing the inactivation ability of GSE in different growth phases; however, a similar sensitivity of exponential cells was observed for other stress factors including natural antimicrobials [78,80,81,82,83,84]. For example, Jydegaard et al. (2000) demonstrated that when *L. monocytogenes* was challenged in liquid nutrient media by nisin (300 IU/mL) or/and pediocin (320 AU/mL), the inactivation was increased in mid-exponential cells in comparison to stationary phase cells. Similarly, it was observed that mid-exponential cells of *S. aureus* had a population reduction of 4 log CFU/mL after 3 h of exposure to liquid nutrient media containing 15 μg/mL of propolis. The inactivation in the late-exponential and stationary phase was much less, i.e., 0.87 and 0.28, respectively [84].

However, as can be seen in Figure 1c, the inactivation dynamics of *L. monocytogenes* cells from mid-exponential phase also showed great variation between samples (Figure 1c). The cause of this high variation can be attributed to the heterogeneity of cells in the mid-exponential phase [85]. A similar variation in the inactivation of exponential phase bacterial cells has been reported for various bacteria and for other stress factors as well [86,87,88,89]. For example, in a study by Coroller et al. (2006), cells in different growth phases of *L. monocytogenes* were subjected to acidic stress in liquid nutrient media containing hydrochloric acid (pH = 3.3). The initial inoculum concentration of *L. monocytogenes* was 10^7^ CFU/mL. Higher variation was observed between replicate experiments for exponential phase cells as compared to stationary phase cells [89]. Furthermore, in a study by Ortuno et al. (2012), when *E. coli* (10^7^ CFU/mL) was treated in liquid nutrient media with supercritical carbon dioxide, the variation in microbial dynamics was much higher in early exponential phase cells in contrast to early stationary phase cells [86]. The magnitude of the variation might depend on the type of treatment. For milder inactivation methods, such as treatment with natural antimicrobials, the variation in inactivation in exponential phase cells could be greater, as during exponential growth low numbers of persistent cells can be formed when exposed to a sublethal dose of antimicrobials [90,91,92]. These cells are a subpopulation resistant to the natural antimicrobial, which can survive the inactivation, while the rest of the cells remain susceptible. Therefore, the growth phase of the bacterial cells is an important factor to be considered when validating decontamination processes, especially as changes in the physiological state of the cells could substantially alter the treatment response.

#### 3.1.3. The Impact of sigB on the Activity of GSE against *L. monocytogenes*

As shown in Section 3.1.2, the growth phase of bacterial cells had a substantial impact on the antimicrobial activity of GSE. As discussed previously, the bacterial growth phase is directly linked to the tolerance of the cell under environmental stresses. The *sigB* gene is partly responsible for the ability of *L. monocytogenes* to survive and adapt through various environmental stresses and is over-expressed during the stationary phase [60,93]. Therefore, to elucidate the role of the environmental stress response and/or survival of *L. monocytogenes* against GSE, further experiments were performed using a mutant of *L. monocytogenes*, in *sigB* (Δ*sigB*). As stated in Section 3.1.1, the level of microbial inactivation for the wild type *L. monocytogenes* was substantial (~3 log CFU/mL reduction) when the initial inoculum concentration was 10^5^ CFU/mL and when the bacterial cells were challenged with 1% GSE. A similar trend was observed at higher inoculum and GSE concentration (10^8^ CFU/mL) for 4% GSE. The same parameters were tested using the mutant Δ*sigB* strain to enable a direct comparison between the mutant and the wild type. Based on our previous results with the wild type (Section 3.1.1 and Section 3.1.2), stationary phase cells were used as (i) they are the most resistant to mild treatments including GSE (ii) their resistance and response is consistent and shows less variability.

**Figure 2:**
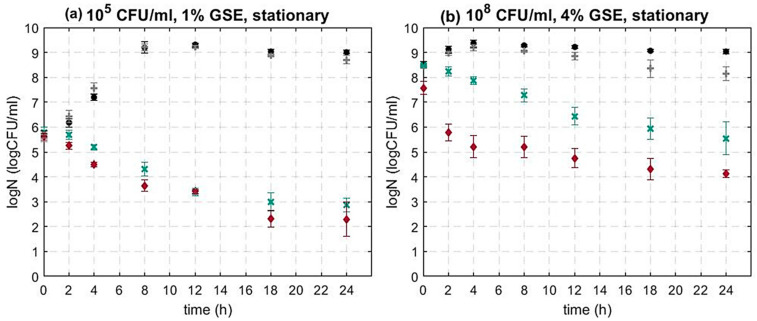
Inactivation dynamics of *L. monocytogenes* 10403S WT and Δ*sigB* at (**a**) initial inoculum concentration 10^5^ CFU/mL and 1% GSE (**b**) initial inoculum concentration 10^8^ CFU/mL and 4% GSE. In both plots (●) control WT (*w/o* GSE), (**+**) control Δ*sigB* (*w/o* GSE), (**×**) treated sample WT with 1% or 4% GSE, (♦) treated sample Δ*sigB* with 1% or 4% GSE. Each time point represents the average of two independent experiments with three technical replicates per experiments. Error bars show standard deviation.

Figure 2a shows the microbial dynamics of the wild type (WT) of *L. monocytogenes* and the *sigB* mutant in the presence of 1% GSE. The microbial population of the mutant decreased significantly more than the WT (*p* < 0.05). The difference in terms of the surviving population between WT and *sigB* mutant was 0.6 log CFU/mL on average. At higher inoculum and GSE concentrations (10^8^ CFU/mL and 4% GSE), the difference was even more evident (Figure 2b).

These results show that the central gene regulator SigB plays a critical role in the response and potential tolerance of *L. monocytogenes* to GSE. *To the authors’ best knowledge there is no other study reporting the effect of SigB on the inactivation of L. monocytogenes in the presence of GSE*. In addition, there are limited studies exploring the impact of SigB on the tolerance of *L. monocytogenes* to other natural antimicrobials and the results are contradictory [63,94]. For example, Begley et al. (2006) reported that SigB contributed to the survival of *L. monocytogenes* when challenged in brain heart infusion broth (BHI) supplemented with nisin and/or lacticin 3417. However, in another study by Palmer et al. (2009), the ΔSigB strain exhibited increased resistance in comparison to the WT when inoculated in BHI that contained nisin [94].

A potential explanation of SigB’s role in the resistance of *L. monocytogenes* to antimicrobials and other stress factors is the regulation of the cell membrane characteristics such as the charge or the lipid composition [63,95]. It has been reported that GSE has the ability to penetrate the bacterial membrane, resulting in cell leakage [47,48,64]. Therefore, the absence of SigB and, thus, the ability to alter the membrane composition as a response to GSE could further decrease the cell tolerance. Alternatively, SigB could potentially control the expression of proteins involved in the expulsion of the antimicrobials from the cell and their absence in the mutant could result in the faster accumulation of GSE in the cell, eventually causing damage and death [63].

The increased sensitivity of ΔSigB in the presence of GSE is particularly interesting as SigB could be an appealing target for the development of novel control strategies against *L. monocytogenes*, which remains one of the most dangerous and fatal foodborne pathogens [70].

### 3.2. The Impact of GSE on the Microbial Dynamics of Gram-Negative Bacteria

Our data described in the previous sections show the great potential of GSE to inactivate the Gram-positive pathogen *L. monocytogenes*. However, to develop wider antimicrobial control strategies, it is of interest to explore whether GSE can act against Gram-negative bacterial pathogens such as *E. coli* and *S.* Typhimurium. In general, Gram-negative bacteria are considered to be less susceptible to natural antimicrobials as compared to Gram-positive bacteria [13,57,96]. There are very limited studies of detailed microbial dynamics of Gram-negative bacteria in an in vitro model using GSE. Most studies have either focused on inactivation in specific food products or assessed inactivation only at one time point post-treatment [28,47,55].

As mentioned previously, in order to apply GSE in food processing, it is crucial to evaluate potential inactivation trends, re-growth or resistance, especially during the initial post-treatment period. To study the effect of GSE on the dynamics of Gram-negative bacteria, *E. coli* and *S.* Typhimurium, 1% and 4% GSE concentrations were added in the growth media, as per the experiments for *L. monocytogenes*. The initial inoculum concentration was equal to 10^5^ CFU/mL and stationary phase cells were used to enable a fair comparison between the different bacteria.

**Figure 3:**
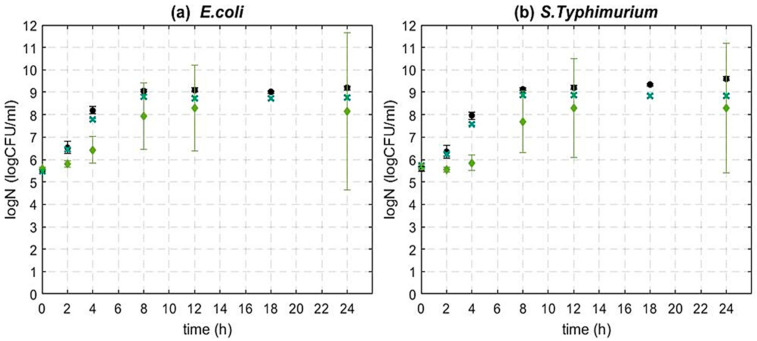
Inactivation dynamics of (**a**) *E. coli* MG1655 and (**b**) *S*. Typhimurium ATCC 14028 stationary phase cells at initial inoculum concentration 10^5^ CFU/mL. In both plots, (●) control (*w*/*o* GSE), (**×**) 1% GSE and (♦) 4% GSE. Each time point represents the average of at least four independent experiments with significant difference (*p* < 0.05) and three technical replicates per experiment. Error bars show standard deviation.

As shown in Figure 3, both Gram-negative bacteria under study showed a higher tolerance to 1% GSE in comparison with *L. monocytogenes* (Figure 1). More specifically, growth was observed for both bacteria in the presence of both concentrations of GSE. However, a disturbance in the growth of *E. coli* and *S.* Typhimurium occurred as compared to untreated controls (Figure 3). More specifically, for *E. coli*, the difference in bacterial concentration between the control and the treated sample (1% GSE) ranged from 0.18 to 0.42 log CFU/mL. Similarly, the population of *S.* Typhimurium treated with 1% of GSE was below the control sample by 0.10–0.78 log CFU/mL, depending on the time point. As the inactivation with 1% GSE was not substantial, the same inoculum concentration (10^5^ CFU/mL) of *E. coli* and *S.* Typhimurium was also challenged with higher concentration of GSE (4%). A significant inhibitory growth effect was shown for 4% GSE at the initial stages of growth (up to 4 h) with the effect becoming statistically insignificant and highly variable between replicate experiments after 4 h of growth for both bacteria under study.

Previously published studies report contradictory results regarding the efficacy of GSE against Gram-negative bacteria [28,47,51,54,55,57,97]. For example, Corrales et al. (2009) performed an agar diffusion test and observed no inhibition effect for either of the Gram-negative bacteria studied, i.e., *E. coli* and *S.* Typhimurium, even at the highest concentration of 20%. The lack of inhibition was attributed to the additional outer lipid membrane existing in Gram-negative bacteria preventing antimicrobial compounds from penetrating the cell wall [47]. Similarly, in another study by Kao et al. (2010), which tested the antimicrobial activity of GSE against *E. coli* and *Stenotrophomonas maltophilia* (Gram-negative), *S. aureus* and *E. faecalis* (Gram-positive) with the agar diffusion test, it was found that Gram-negative bacteria were more tolerant to GSE as compared to Gram positive, which is similar to our findings [57]. In contrast, Ahn et al. (2004) challenged *E. coli* and *S.* Typhimurium at inoculum concentration 5 × 10^4^ CFU/mL in liquid nutrient media containing 1% GSE and observed that the decrease in bacterial count was substantial (>3 log CFU/mL) after 12 h of treatment. Later (2007), the same research group studied the effect of GSE (1%) on the microbial kinetics of *E. coli*, *S.* Typhimurium, *Aeromonas hydrophila* and *L. monocytogenes* in cooked beef during storage (4 °C). A reduction of 1 log (in comparison with the control) was detected in both Gram-negative and Gram-positive bacteria after 9 d of storage [55]. Furthermore, Baydar et al. (2006) used seed extracts of three different grape variants at 1% concentration to challenge *E. coli* and *S. aureus* (10^7^ CFU/mL) in liquid nutrient media, and observed a 1.5 log CFU/mL reduction for *E. coli* and an almost total inhibition for *S. aureus* after 24 h [51].

Our results indicate that 1% and 4% of GSE have little to no effect on the kinetics of the Gram-negative bacteria, *E. coli* and *S.* Typhimurium in nutrient broth at 37 °C (the optimal growth temperature for those bacteria). Therefore, for the efficient inactivation of these bacteria with GSE, a hurdle approach might be required combining GSE with other mild treatments, similarly to hurdle approaches reported to enhance the activity of other antimicrobials such as nisin [3,73,75,98].

## 4. Conclusions

A systematic study of the microbial inactivation efficacy/potential of grape seed extract (GSE) against *L. monocytogenes* (Gram-positive), *E. coli* and *S.* Typhimurium (Gram-negative) was carried out in an in vitro model system. More specifically, for *L. monocytogenes*, the effects of the initial inoculum concentration, bacterial growth phase and absence of the environmental stress response regulon (SigB) on the GSE microbial inactivation potential were investigated. In general, GSE was found to be highly effective at inactivating *L. monocytogenes*. Stronger inactivation was achieved with higher GSE concentrations and lower initial inoculum levels. Generally, stationary phase cells were more resistant/tolerant to GSE as compared to exponential phase cells at the same inoculum level. Additionally, SigB appears to play an important role in the resistance of *L. monocytogenes* to GSE. The absence of SigB resulted in a higher inactivation potential of GSE against *L. monocytogenes.* Finally, Gram-negative bacteria (*E. coli* and *S.* Typhimurium) exhibited greater resistance to GSE as compared to *L. monocytogenes*. Our findings shine more light on the potential of GSE as a sustainable antimicrobial strategy in the food industry as well as on parameters that can affect its efficiency against common food-related pathogens.

Future work should focus on combinatory treatments to further challenge and eventually inactivate Gram-negative bacteria which appeared to be more resistant to the use of GSE. An example of such a treatment is the use of GSE in combination with other mild/non-optimal treatments and/or novel technologies such as ultrasound or cold atmospheric plasma.

## Figures and Tables

**Table 1:** Parameters tested in the experiments.

Microorganism	Growth Phase	Initial Population (CFU/mL)	GSE Concentration (*w/v*)
*L. monocytogenes* 10403S WT	Stationary	10^5^	1%
		10^8^	1% and 4%
	Mid-exponential	10^5^	1%
*L. monocytogenes* 10403S Δ*sigB*	Stationary	10^5^	1%
		10^8^	4%
*E. coli* MG1655	Stationary	10^5^	1% and 4%
*S.* Typhimurium ATCC 14028	Stationary	10^5^	1% and 4%

## Data Availability

The datasets generated for this study are available on reasonable request to the corresponding author.

## References

[1] Arenas-Jal M., Suñé-Negre J.M., Pérez-Lozano P., García-Montoya E. (2020). Trends in the food and sports nutrition industry: A review. Crit. Rev. Food Sci. Nutr..

[2] Zhang Z.-H., Wang L.-H., Zeng X.-A., Han Z., Brennan C.S. (2019). Non-thermal technologies and its current and future application in the food industry: A review. Int. J. Food Sci. Technol..

[3] Costello K.M., Gutierrez-Merino J., Bussemaker M., Smet C., Van Impe J.F., Velliou E.G. (2019). A multi-scale analysis of the effect of complex viscoelastic models on *Listeria* dynamics and adaptation in co-culture systems. AIChE J..

[4] Goryluk-Salmonowicz A., Popowska M. (2022). Factors promoting and limiting antimicrobial resistance in the environment—Existing knowledge gaps. Front. Microbiol..

[5] EFSA (European Food Safety Authority), ECDC (European Centre for Disease Prevention and Control) (2017). The European Union summary report on antimicrobial resistance in zoonotic and indicator bacteria from humans, animals and food in 2017. EFSA J..

[6] Landers T.F., Cohen B., Wittum T.E., Larson E.L. (2012). A Review of Antibiotic Use in Food Animals: Perspective, Policy, and Potential. Public Health Rep..

[7] Ma F., Xu S., Tang Z., Li Z., Zhang L. (2021). Use of antimicrobials in food animals and impact of transmission of antimicrobial resistance on humans. Biosaf. Health.

[8] Michael C.A., Dominey-Howes D., Labbate M. (2014). The Antimicrobial Resistance Crisis: Causes, Consequences, and Management. Front. Public Health.

[9] O’Neill J. (2016). Tackling Drug-Resistant Infections Globally: Final Report and Recommendations.

[10] Teixeira J.A. (2018). Grand Challenges in Sustainable Food Processing. Front. Sustain. Food Syst..

[11] Searchinger T., Hanson C., Ranganathan J., Lipinski B., Waite R., Winterbottom R., Dinshaw A., Heimlich R. (2013). Creating a Sustainable Food Future: Interim Findings. A Menu of Solutions to Sustainably Feed More than 9 Billion People by 2050.

[12] Liu X., Liu R., Zhao R., Wang J., Cheng Y., Liu Q., Wang Y., Yang S. (2022). Synergistic Interaction Between Paired Combinations of Natural Antimicrobials Against Poultry-Borne Pathogens. Front. Microbiol..

[13] Quinto E.J., Caro I., Villalobos-Delgado L.H., Mateo J., De-Mateo-Silleras B., Redondo-Del-Río M.P. (2019). Food Safety through Natural Antimicrobials. Antibiotics.

[14] Villalobos-Delgado L.H., Nevárez-Moorillon G.V., Caro I., Quinto E.J., Mateo J. (2019). Natural antimicrobial agents to improve foods shelf life. Food Quality and Shelf Life, Galanakis.

[15] Gómez N.C., Manetsberger J., Benomar N., Gutiérrez S.C., Abriouel H. (2022). Antibacterial and antibiofilm effects of essential oil components, EDTA and HLE disinfectant solution on Enterococcus, Pseudomonas and Staphylococcus sp. multiresistant strains isolated along the meat production chain. Front. Microbiol..

[16] Sabater C., Ruiz L., Delgado S., Ruas-Madiedo P., Margolles A. (2020). Valorization of Vegetable Food Waste and By-Products through Fermentation Processes. Front. Microbiol..

[17] Idris S., Ndukwe G., Gimba C. (2010). Preliminary phytochemical screening and antimicrobial activity of seed extracts of Persea americana (avocado pear). Bayero J. Pure Appl. Sci..

[18] Al-Zoreky N. (2009). Antimicrobial activity of pomegranate (*Punica granatum* L.) fruit peels. Int. J. Food Microbiol..

[19] Andrade M.A., Lima V., Silva A.S., Vilarinho F., Castilho M.C., Khwaldia K., Ramos F. (2019). Pomegranate and grape by-products and their active compounds: Are they a valuable source for food applications?. Trends Food Sci. Technol..

[20] Agourram A., Ghirardello D., Rantsiou K., Zeppa G., Belviso S., Romane A., Oufdou K., Giordano M. (2013). Phenolic Content, Antioxidant Potential, and Antimicrobial Activities of Fruit and Vegetable By-Product Extracts. Int. J. Food Prop..

[21] Abdalla A.E., Darwish S.M., Ayad E.H., El-Hamahmy R.M. (2007). Egyptian mango by-product 2: Antioxidant and antimicrobial activities of extract and oil from mango seed kernel. Food Chem..

[22] Saleem M., Saeed M.T. (2019). Potential application of waste fruit peels (orange, yellow lemon and banana) as wide range natural antimicrobial agent. J. King Saud Univ. Sci..

[23] Pandey K.B., Rizvi S.I. (2009). Plant polyphenols as dietary antioxidants in human health and disease. Oxid. Med. Cell. Longev..

[24] Daglia M. (2012). Polyphenols as antimicrobial agents. Curr. Opin. Biotechnol..

[25] Chedea V.S., Pop R.M. (2019). Total Polyphenols Content and Antioxidant DPPH Assays on Biological Samples.

[26] Martin M.E., Grao-Cruces E., Millan-Linares M.C., Montserrat de la Paz S. (2020). Grape (*Vitis vinifera* L.) Seed Oil: A Functional Food from the Winemaking Industry. Foods.

[27] Oliveira D.A., Salvador A.A., Smânia A., Smânia E.F., Maraschin M., Ferreira S.R. (2012). Antimicrobial activity and composition profile of grape (*Vitis vinifera*) pomace extracts obtained by supercritical fluids. J. Biotechnol..

[28] Özkan G., Sagdiç O., Baydar N.G., Kurumahmutoglu Z. (2004). Antibacterial activities and total phenolic contents of grape pomace extracts. J. Sci. Food Agric..

[29] Anastasiadi M., Chorianopoulos N.G., Nychas G.-J.E., Haroutounian S.A. (2008). Antilisterial Activities of Polyphenol-Rich Extracts of Grapes and Vinification Byproducts. J. Agric. Food Chem..

[30] Farhadi K., Esmaeilzadeh F., Hatami M., Forough M., Molaie R. (2016). Determination of phenolic compounds content and antioxidant activity in skin, pulp, seed, cane and leaf of five native grape cultivars in West Azerbaijan province, Iran. Food Chem..

[31] Ribeiro L.F., Ribani R.H., Francisco T.M.G., Soares A.A., Pontarolo R., Haminiuk C.W.I. (2015). Profile of bioactive compounds from grape pomace (*Vitis vinifera* and *Vitis labrusca*) by spectrophotometric, chromatographic and spectral analyses. J. Chromatogr. B.

[32] Zhao X., Chen L., Wu J., He Y., Yang H. (2019). Elucidating antimicrobial mechanism of nisin and grape seed extract against Listeria monocytogenes in broth and on shrimp through NMR-based metabolomics approach. Int. J. Food Microbiol..

[33] FDA (2003). Grape Seed Extract (GSE) Notification.

[34] Xu Y., Burton S., Kim C., Sismour E. (2016). Phenolic compounds, antioxidant, and antibacterial properties of pomace extracts from four Virginia-grown grape varieties. Food Sci. Nutr..

[35] Yu J., Ahmedna M. (2013). Functional components of grape pomace: Their composition, biological properties and potential applications. Int. J. Food Sci. Technol..

[36] Lingua M.S., Fabani M.P., Wunderlin D.A., Baroni M.V. (2016). In vivo antioxidant activity of grape, pomace and wine from three red varieties grown in Argentina: Its relationship to phenolic profile. J. Funct. Foods.

[37] Goula A., Thymiatis K., Kaderides K. (2016). Valorization of grape pomace: Drying behavior and ultrasound extraction of phenolics. Food Bioprod. Process..

[38] Brewer M.S. (2011). Natural Antioxidants: Sources, Compounds, Mechanisms of Action, and Potential Applications. Compr. Rev. Food Sci. Food Saf..

[39] Drosou C., Kyriakopoulou K., Bimpilas A., Tsimogiannis D., Krokida M. (2015). A comparative study on different extraction techniques to recover red grape pomace polyphenols from vinification byproducts. Ind. Crop. Prod..

[40] Sagdic O., Ozturk I., Yilmaz M.T., Yetim H. (2011). Effect of Grape Pomace Extracts Obtained from Different Grape Varieties on Microbial Quality of Beef Patty. J. Food Sci..

[41] Lončarević I., Petrović J., Teslić N., Nikolić I., Maravić N., Pajin B., Pavlić B. (2022). Cocoa Spread with Grape Seed Oil and Encapsulated Grape Seed Extract: Impact on Physical Properties, Sensory Characteristics and Polyphenol Content. Foods.

[42] Silva V., Igrejas G., Falco V., Santos T.P., Torres C., Oliveira A.M., Pereira J.E., Amaral J.S., Poeta P. (2018). Chemical composition, antioxidant and antimicrobial activity of phenolic compounds extracted from wine industry by-products. Food Control..

[43] Katalinić V., Možina S.S., Skroza D., Generalić I., Abramovič H., Miloš M., Ljubenkov I., Piskernik S., Pezo I., Terpinc P. (2010). Polyphenolic profile, antioxidant properties and antimicrobial activity of grape skin extracts of 14 Vitis vinifera varieties grown in Dalmatia (Croatia). Food Chem..

[44] Adámez J.D., Samino E.G., Sánchez E.V., González-Gómez D. (2012). In vitro estimation of the antibacterial activity and antioxidant capacity of aqueous extracts from grape-seeds (*Vitis vinifera* L.). Food Control..

[45] Baydar N.G., Özkan G., Sagdic O. (2004). Total phenolic contents and antibacterial activities of grape (*Vitis vinifera* L.) extracts. Food Control..

[46] Silvan J.M., Gutiérrez-Docio A., Moreno-Fernandez S., Alarcón-Cavero T., Prodanov M., Martinez-Rodriguez A.J. (2020). Procyanidin-Rich Extract from Grape Seeds as a Putative Tool against *Helicobacter pylori*. Foods.

[47] Corrales M., Han J.H., Tauscher B. (2009). Antimicrobial properties of grape seed extracts and their effectiveness after incorporation into pea starch films. Int. J. Food Sci. Technol..

[48] Silván J.M., Mingo E., Hidalgo M., de Pascual-Teresa S., Carrascosa A.V., Martinez-Rodriguez A.J. (2013). Antibacterial activity of a grape seed extract and its fractions against *Campylobacter* spp.. Food Control..

[49] Begg S.L. (2019). The role of metal ions in the virulence and viability of bacterial pathogens. Biochem. Soc. Trans..

[50] Klančnik A., Piskernik S., Jeršek B., Možina S.S. (2010). Evaluation of diffusion and dilution methods to determine the antibacterial activity of plant extracts. J. Microbiol. Methods.

[51] Baydar N.G., Sagdic O., Ozkan G., Cetin S. (2006). Determination of antibacterial effects and total phenolic contents of grape (*Vitis vinifera* L.) seed extracts. Int. J. Food Sci. Technol..

[52] Sivarooban T., Hettiarachchy N.S., Johnson M.G. (2007). Inhibition of *Listeria monocytogenes* using nisin with grape seed extract on turkey frankfurters stored at 4 and 10 °C. J. Food Prot..

[53] Zhao X., Chen L., Zhao L., He Y., Yang H. (2020). Antimicrobial kinetics of nisin and grape seed extract against inoculated Listeria monocytogenes on cooked shrimps: Survival and residual effects. Food Control..

[54] Ahn J., Grün I.U., Mustapha A. (2004). Antimicrobial and Antioxidant Activities of Natural Extracts in Vitro and in Ground Beef. J. Food Prot..

[55] Ahn J., Grün I.U., Mustapha A. (2007). Effects of plant extracts on microbial growth, color change, and lipid oxidation in cooked beef. Food Microbiol..

[56] Sheng L., Olsen S.A., Hu J., Yue W., Means W.J., Zhu M.J. (2016). Inhibitory effects of grape seed extract on growth, quorum sensing, and virulence factors of CDC “top-six” non-O157 Shiga toxin producing *E. coli*. Int. J. Food Microbiol..

[57] Kao T.-T., Tu H.-C., Chang W.-N., Chen B.-H., Shi Y.-Y., Chang T.-C., Fu T.-F. (2010). Grape seed extract inhibits the growth and pathogenicity of Staphylococcus aureus by interfering with dihydrofolate reductase activity and folate-mediated one-carbon metabolism. Int. J. Food Microbiol..

[58] Pletnev P., Osterman I., Sergiev P.V., Bogdanov A., Dontsova O. (2015). Survival guide: *Escherichia coli* in the stationary phase. Acta Nat..

[59] Utratna M., Cosgrave E., Baustian C., Ceredig R.H., O’Byrne C.P. (2014). Effects of Growth Phase and Temperature on σB Activity within a *Listeria monocytogenes* Population: Evidence for RsbV-Independent Activation of σB at Refrigeration Temperatures. BioMed Res. Int..

[60] Boura M., Keating C., Royet K., Paudyal R., O’Donoghue B., O’Byrne C.P., Karatzas K.A.G. (2016). Loss of SigB in Listeria monocytogenes Strains EGD-e and 10403S Confers Hyperresistance to Hydrogen Peroxide in Stationary Phase under Aerobic Conditions. Appl. Environ. Microbiol..

[61] O’Byrne C.P., Karatzas K.A. (2008). The Role of Sigma B (σB) in the Stress Adaptations of Listeria monocytogenes: Overlaps between Stress Adaptation and Virulence. Adv. Appl. Microbiol..

[62] Raengpradub S., Wiedmann M., Boor K.J. (2008). Comparative Analysis of the σ ^B^ -Dependent Stress Responses in *Listeria monocytogenes* and *Listeria innocua* Strains Exposed to Selected Stress Conditions. Appl. Environ. Microbiol..

[63] Begley M., Hill C., Ross R.P. (2006). Tolerance of *Listeria monocytogenes* to Cell Envelope-Acting Antimicrobial Agents Is Dependent on SigB. Appl. Environ. Microbiol..

[64] Sivarooban T., Hettiarachchy N.S., Johnson M.G. (2008). Transmission electron microscopy study of *Listeria monocytogenes* treated with nisin in combination with either grape seed or green tea extract. J. Food Prot..

[65] Fong I.W., Engelking E.R., Kirby W.M.M. (1976). Relative Inactivation by *Staphylococcus aureus* of Eight Cephalosporin Antibiotics. Antimicrob. Agents Chemother..

[66] Bacon R.T., Ransom J.R., Sofos J.N., Kendall P.A., Belk K.E., Smith G.C. (2003). Thermal Inactivation of Susceptible and Multiantimicrobial-Resistant *Salmonella* Strains Grown in the Absence or Presence of Glucose. Appl. Environ. Microbiol..

[67] Shen C., Sofos J. (2008). Antilisterial Activity of Hops Beta Acids in Broth with or Without Other Antimicrobials. J. Food Sci..

[68] Oh D.-H., Marshall D.L. (1993). Antimicrobial activity of ethanol, glycerol monolaurate or lactic acid against Listeria monocytogenes. Int. J. Food Microbiol..

[69] Lambert R., Skandamis P., Coote P., Nychas G.-J. (2001). A study of the minimum inhibitory concentration and mode of action of oregano essential oil, thymol and carvacrol. J. Appl. Microbiol..

[70] European Food Safety Authority (2021). European Centre for Disease Prevention and Control The European Union One Health 2020 Zoonoses Report. EFSA J..

[71] Velliou E., Van Derlinden E., Cappuyns A., Geeraerd A., Devlieghere F., Van Impe J. (2012). Heat inactivation of *Escherichia coli* K12 MG1655: Effect of microbial metabolites and acids in spent medium. J. Therm. Biol..

[72] Velliou E., Noriega E., Van Derlinden E., Mertens L., Boons K., Geeraerd A., Devlieghere F., Van Impe J. (2013). The effect of colony formation on the heat inactivation dynamics of *Escherichia coli* K12 and Salmonella typhimurium. Food Res. Int..

[73] Costello K.M., Smet C., Gutierrez-Merino J., Bussemaker M., Van Impe J.F., Velliou E.G. (2021). The impact of food model system structure on the inactivation of Listeria innocua by cold atmospheric plasma and nisin combined treatments. Int. J. Food Microbiol..

[74] Velliou E., Van Derlinden E., Cappuyns A., Aerts D., Nikolaidou E., Geeraerd A., Devlieghere F., Van Impe J. (2010). Quantification of the influence of trimethylamine-N-oxide (TMAO) on the heat resistance of *Escherichia coli* K12 at lethal temperatures. Lett. Appl. Microbiol..

[75] Costello K.M., Gutierrez-Merino J., Bussemaker M., Ramaioli M., Baka M., Van Impe J.F., Velliou E.G. (2018). Modelling the microbial dynamics and antimicrobial resistance development of Listeria in viscoelastic food model systems of various structural complexities. Int. J. Food Microbiol..

[76] Velliou E.G., Van Derlinden E., Cappuyns A.M., Goossens J., Geeraerd A.H., Devlieghere F., Van Impe J.F. (2011). Heat adaptation of *Escherichia coli* K12: Effect of acid and glucose. Procedia Food Sci..

[77] Velliou E., Van Derlinden E., Cappuyns A., Nikolaidou E., Geeraerd A., Devlieghere F., Van Impe J. (2011). Towards the quantification of the effect of acid treatment on the heat tolerance of *Escherichia coli* K12 at lethal temperatures. Food Microbiol..

[78] Jydegaard A.-M., Gravesen A., Knøchel S. (2000). Growth condition-related response of *Listeria monocytogenes* 412 to bacteriocin inactivation. Lett. Appl. Microbiol..

[79] Jaishankar J., Srivastava P. (2017). Molecular Basis of Stationary Phase Survival and Applications. Front. Microbiol..

[80] Davis M.J., Coote P.J., O’Byrne C.P. (1996). Acid tolerance in Listeria monocytogenes: The adaptive acid tolerance response (ATR) and growth-phase-dependent acid resistance. Microbiology.

[81] Mendonça A.F., Daraba A. (2014). NON-THERMAL PROCESSING|Irradiation. Encyclopedia of Food Microbiology.

[82] Mañas P., Mackey B.M. (2004). Morphological and Physiological Changes Induced by High Hydrostatic Pressure in Exponential- and Stationary-Phase Cells of *Escherichia coli*: Relationship with Cell Death. Appl. Environ. Microbiol..

[83] Cebrián G., Sagarzazu N., Pagán R., Condón S., Mañas P. (2007). Heat and pulsed electric field resistance of pigmented and non-pigmented enterotoxigenic strains of Staphylococcus aureus in exponential and stationary phase of growth. Int. J. Food Microbiol..

[84] Lu L.-C., Chen Y.-W., Chou C.-C. (2005). Antibacterial activity of propolis against Staphylococcus aureus. Int. J. Food Microbiol..

[85] Rocco A., Kierzek A.M., McFadden J. (2013). Slow Protein Fluctuations Explain the Emergence of Growth Phenotypes and Persistence in Clonal Bacterial Populations. PLoS ONE.

[86] Ortuño C., Martínez-Pastor M.T., Mulet A., Benedito J. (2012). Supercritical carbon dioxide inactivation of *Escherichia coli* and Saccharomyces cerevisiae in different growth stages. J. Supercrit. Fluids.

[87] Zhao L., Qin X., Wang Y., Ling J., Shi W., Pang S., Liao X. (2017). CO_2_ -assisted high pressure processing on inactivation of *Escherichia coli* and Staphylococcus aureus. J. CO2 Util..

[88] Yu H., Perni S., Shi J., Wang D., Kong M., Shama G. (2006). Effects of cell surface loading and phase of growth in cold atmospheric gas plasma inactivation of *Escherichia coli* K12. J. Appl. Microbiol..

[89] Coroller L., Leguerinel I., Mettler E., Savy N., Mafart P. (2006). General Model, Based on Two Mixed Weibull Distributions of Bacterial Resistance, for Describing Various Shapes of Inactivation Curves. Appl. Environ. Microbiol..

[90] Kearns D.B., Losick R. (2005). Cell population heterogeneity during growth of *Bacillus subtilis*. Genes Dev..

[91] Schottroff F., Fröhling A., Zunabovic-Pichler M., Krottenthaler A., Schlüter O., Jäger H. (2018). Sublethal Injury and Viable but Non-culturable (VBNC) State in Microorganisms During Preservation of Food and Biological Materials by Non-thermal Processes. Front. Microbiol..

[92] Wu S., Yu P.-L., Flint S. (2017). Persister cell formation of Listeria monocytogenes in response to natural antimicrobial agent nisin. Food Control..

[93] Dorey A.L., Lee B.-H., Rotter B., O’Byrne C.P. (2019). Blue Light Sensing in Listeria monocytogenes Is Temperature-Dependent and the Transcriptional Response to It Is Predominantly SigB-Dependent. Front. Microbiol..

[94] Palmer M.E., Wiedmann M., Boor K.J. (2009). σ^B^ and σ^L^ Contribute to *Listeria monocytogenes* 10403S Response to the Antimicrobial Peptides SdpC and Nisin. Foodborne Pathog. Dis..

[95] Guerreiro D.N., Arcari T., O’Byrne C.P. (2020). The σB-Mediated General Stress Response of Listeria monocytogenes: Life and Death Decision Making in a Pathogen. Front. Microbiol..

[96] Gyawali R., Ibrahim S.A. (2014). Natural products as antimicrobial agents. Food Control..

[97] Deng Q., Zhao Y. (2011). Physicochemical, Nutritional, and Antimicrobial Properties of Wine Grape (cv. Merlot) Pomace Extract-Based Films. J. Food Sci..

[98] Costello K.M., Velliou E., Gutierrez-Merino J., Smet C., El Kadri H., Van Impe J.F., Bussemaker M. (2021). The effect of ultrasound treatment in combination with nisin on the inactivation of *Listeria innocua* and *Escherichia coli*. Ultrason. Sonochemistry.

